# The Effect of Sex on the Electropsychological Process of Emotional Arousal Intensity

**DOI:** 10.21315/mjms2018.25.3.10

**Published:** 2018-06-28

**Authors:** Nasir Yusoff, Nik NurAzhani Anuar, Mohammed Faruque Reza

**Affiliations:** Department of Neurosciences, School of Medical Sciences, Universiti Sains Malaysia, Health Campus, 16150 Kubang Kerian, Kelantan, Malaysia

**Keywords:** sex, evoked potentials, P300 components, emotion, emotional arousal

## Abstract

**Background:**

Sex is a psychobiological factor that is important in the process of emotion. This study determines the effect of sex on the electropsychological process of various intensities of emotional arousal.

**Methods:**

In the Event-related Potential (ERP) session, electroencephalographic (EEG) data was recorded for 90 participants, 60% of whom were females. The participants responded to 30 universal emotional pictures, randomly chosen from the International Affective Picture System (IAPS), which were classified as invoking high, moderate, and low intensity of emotional arousal.

**Results:**

From the analysis of variance of two-way mixed design, the interaction between sex and emotional intensity was observed in the occipital regions (O2), indexed by the amplitude of P300 and N200 components. Males exhibited higher amplitude of P300 and N200 components (in the occipital region) as responded to high and low emotional arousal stimuli than females.

**Conclusion:**

Sex is a fundamental factor that modulates psychological states in reaction to emotional stimuli.

## Introduction

The effect of sex on psychological and physiological factors have been clearly documented (1–6). In response to emotional image, females indicated higher negative emotion related to subjective rating, than males. Males were found to indicate significantly different emotional responses especially for the high negative condition, and these responses were reflected by the greater connectivity in the right amygdala than in the dorsomedial prefrontal cortex (7).

In a study in which participants viewed pictures with various emotional and neutral content, males had less defensive reactivity to aversive images and exhibited obvious appetitive activations in response to erotic images than females (8). Indeed, this psychophysiological investigation of emotional intensity that used ‘pleasant’ and ‘unpleasant’ pictures found that, emotional arousal and valence were modulated differently by the sexes. Females were more modulated by emotional arousal associated with a late positive complex, and males were more modulated by valence associated with the early time components of N200 and P300 at the frontal and parietal areas of the brain, respectively (8).

Another psychophysiological study found that females had a greater positivity to unpleasant images than pleasant images, unlike males, who had a greater positivity to pleasant images than neutral images (9). Similarly, a study of emotion used an Event-related Potential (ERP) technique to observe the process of emotion in regards to empathy-evoking pictures, discovered that females had increased visual ERP component of N200 amplitude, and males did not (10).

The importance of the difference between male and female in the electropsychological process of emotion, focusing on the element of arousal and its intensity, is being emphasised in this study. The influence of sex differences on emotional arousal intensity is explained by the effect of the interaction (analysis of variance of two-way mixed design) between these two variables. Thus, is it postulated that, sex has a significant effect (significant interaction) on the process of emotional arousal, as indexed by the amplitude and latency of the visual components of Event-related Potential i.e. P300 and N200.

## Materials and Methods

### Subjects

A total of 90 volunteers (60% female, 56% Chinese) participated in this study. Those with abnormal or uncorrected to normal vision and those who had a history of affective disorder were not selected, and each participant gave informed consent prior to participating in the study. The participants’ mean age was 22.51 (±1.96) years, and 88% of the participants had completed a bachelor’s degree. Moreover, all participants were right handed, and 40% were left-eye dominant. More than half (73%) had a vision problem that had been corrected by glasses or contact lenses.

### Procedure

Each participant was asked to view 30 pictures passively that invoked different intensities of emotional arousal during an electroencephalogram (EEG) recording session. As reaction time and other behavioural measures were not being studied, participants were not instructed to press a key on a keyboard for the study, i.e., engage in a passive procedure. The EEG data of the emotion modulation was captured with a 128 HydroCel GSN connected to a high-input impedance Net Amps 300 amplifier. The electrode impedance was kept to below 50 kΩ, and the data was digitised at 250 Hz.

The visual stimuli (emotion-inducing pictures) was randomly selected from the International Affective Picture System (IAPS) (11) and installed in E-Prime 2.0 software with 30 trials were allocated to each experimental block. The pictures were grouped into three categories based on the normative mean value of IAPS for arousal domain (high = scores of 7–9, moderate = score of 4–6, low = scores of 1–3) (11–12). There were 10 high arousal pictures, 10 moderate arousal pictures, and 10 low arousal pictures. The participants were not aware of the categories of the pictures.

**Figure 1:**
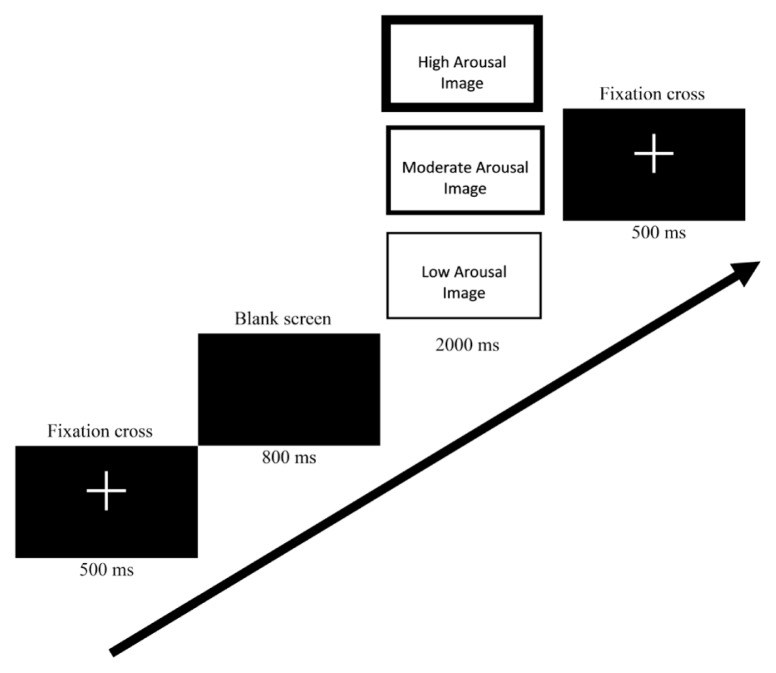
A schematic of the experimental procedure

The IAPS images appeared 2,000 ms after the offset of a fixation mark. The intervals between the offsets of the IAPS images and following fixation marks were 800 ms. Each image was presented in three blocks for a total of 90 test trials. A schematic of the experimental procedure is depicted in Figure 1.

The study was held at the Neuroscience Laboratory in the Hospital of the Universiti Sains Malaysia and was endorsed by the Human Ethical Committee of the Universiti Sains Malaysia (reference number: USM/ JEPeM/15040127).

### Outcome Measure

The extraction of ERP components (P300 and N200 data) was done using EGI Net Station 5.3 software. A 0.30–50.00 Hz bandpass filter was applied, segmentation was locked to 100 ms before the stimuli onset, and segmentation was locked to 1,000 ms after the stimuli onset, with a 17 ms offset. Ocular artefacts (eye blinks and eye movements) and other movement artefacts were removed, and bad channels were recognised once an amplitude difference of 400 μV or larger was identified in a segment. Separate averaging of the data was conducted to increase a signal-to-noise ratio; this was followed by the conversion of the data, which involved a correction from the baseline to a 10–20 EEG montage. Subsequently, the data was combined and averaged to mask subject variability. Then, N200 amplitudes and latencies (a time window of 220–~300 ms) and P300 amplitudes and latencies (a time window of 330–~460 ms) were measured and analysed. Electrode sites in main region (mid-parietal, central, and frontal) and occipital site (O1 and O2) were selected, as these electrodes provided strong evidence of the effects of visualisation on the process of emotion (13–14).

### Statistical Analysis

The analysis of variance of two-way mixed design from the Statistical Package for the Social Sciences (SPSS) Version 23 (Armonk, NY: IBM Corp) was employed to determine the effect of sex (between-subjects effect: male and female) and arousal intensity (within-subjects effect: low, moderate and high arousal) on the electropsychological process of emotional arousal, indexed by the amplitude and latency of the ERP components (P300, N200).

## Results

### P300 Component

Significant interaction effect of sex and arousal intensity was observed in the occipital region (O2), indexed by the amplitude of P300 [*F*(1.71, 150.24) = 3.43, *P* < 0.05]. Male participants indicated higher mean for P300 amplitude (high arousal: mean = 6.2, SD = 4.0; low arousal: mean = 5.8, SD = 3.1), as compared to female participants (high arousal: mean = 5.6, SD = 3.6; low arousal: mean = 5.4, SD = 3.2). However, the main effect of arousal intensity was not detected [*F*(1.71, 150.24) = 0.65, ns] (Table 1).

Although the interaction effects of the P300 latency of sex and arousal intensity were not observed in the mid central (Cz) [*F*(2.0, 87.0) = 0.02, ns] and mid parietal (Pz) area [*F*(2.0, 87.0) = 0.49, ns], however, the main effects were found significant in these areas [Cz: *F*(2.0, 87.0) = 4.24, *P* < 0.05 and Pz: *F*(2.0, 87.0) = 5.62, *P* < 0.01]. Further analysis of pairwise comparison by using Bonferroni method, for the main effect of arousal intensity, indicated the significant difference of high arousal (mean = 583.80; SE = 10.68) versus low arousal (mean = 543.04; SE = 12.00) in the mid central region (mean difference = 40.76, *P* < 0.05; 95% CI = 6.12–75.37). This pattern was similar in the mid parietal region in which the difference was observed between high arousal (mean = 476.30, SE = 12.52) and low arousal (mean = 425.20, SE = 11.70) (mean difference = 51.06, *P* < 0.01; 95% CI = 13.97–88.14).

However, no significant differences were noted in the occiptal (O1) and frontal (Fz) region of the brain, between male and female participants.

### N200 Component

The interaction effect of sex and arousal intensity in the occipital region (O2) was found significant, indexed by the amplitude of N200 [*F*(2.0, 87.0) = 4.0, *P* < 0.05] (Table 2). Again, similar to P300 component, male participants indicated higher mean for N200 amplitude (high arousal: mean = 6.8, SD = 4.2; low arousal: mean = 6.6, SD = 4.5), as compared to female participants (high arousal: mean = 6.1, SD = 3.6; low arousal: mean = 6.2, SD = 3.6). However, the main effect of arousal intensity was not significant [*F*(2.0, 87.0) = 0.18, ns].

Although the interaction effects of the N200 latency of gender and arousal intensity were not observed in the mid central (Cz) [*F*(1.9, 169.0) = 2.14, ns] and mid parietal (Pz) area [F(2.0, 87.0) = 0.83, ns], however, the main effects were found significant in these areas [Cz: *F*(1.9, 169.0) = 10.44, *P* < 0.001 and Pz: *F*(2.0, 87.0) = 3.70, *P* < 0.05]. Pairwise comparison of Bonferroni method was used to explore the differences exist between the categories of arousal intensities. A similar patterns were observed between high arousal (mean = 313.65, SE = 5.52) and low arousal (mean = 286.35, SE = 5.22) in the mid central region (mean difference = 27.30, *P* < 0.001; 95%CI = 11.51–43.09) and between high arousal (mean = 301.50, SE = 6.49) and low arousal (mean = 281.72, SE = 6.52) in the mid parietal region (mean difference = 19.78, *P* < 0.05; 95%CI = 2.08–37.48). In addition, the latency of N200 also indicated the significant difference between high arousal (mean = 301.50, SE = 6.49) and moderate arousal (mean = 294.33, SE = 5.22) in the mid central region (mean difference = 19.32, *P* < 0.05; 95%CI = 3.15–35.48).

Nonetheless, occipital region (O1) and frontal area (Fz) did not exhibit any significant effects in regards to the factor of sex.

## Discussion

The effect of sex on the electropsychological process of emotional arousal is discussed in this section, as well as the main effect of emotional arousal intensity. In this study, sex was found to have a significant effect on the electropsychological process of emotional arousal, as indicated by the ERP components of P300 and N200 amplitude, especially in the occipital region (O2). Moreover, independent of sex, the intensity of arousal was robust mainly in the mid central (Cz) and mid parietal (Pz) area of the brain, as indexed by the latency of P300 and N200.

The effect of sex indeed, could be predicted especially in the electropsychological studies that used emotional pictures to evoke emotions. For example, notable difference between male and female was found that related to left prefrontal region. Females had a greater positivity to unpleasant images than to pleasant images, unlike male subjects (9). Moreover, a functional magnetic resonance imaging study of cognitive reappraisals that examined sex differences related to emotion regulations found that males experienced more neuroanatomical effects related to reappraisal and reward processing than females, especially at the amygdala, prefrontal and ventral striatal regions. Additionally, a study found that males had greater use of automatic emotion regulation than females (15). These findings could explain the present study’s data, which showed male had higher mean of P300 and N200 amplitudes at the occipital region (O2) than female, especially in regards to the processes of high- and low-intensity emotional arousal.

Our finding was supported by several previous important findings. For example, a study of a large sample of young adults (*n* = 1,080) found sex differences in regards to cognitive emotion-regulation strategies, especially in regards to rumination, perspective gaining and blaming (16). It was reported that males used fewer emotion regulation strategies than females (17). Sex’s effect on such strategies was particularly noted in regards to emotion regulation difficulties; males had a tendency to be inhibited, a dominant response that was connected to low emotion regulation difficulties. In contrast, females had great emotion regulation difficulties as a result of their executive functioning capabilities (18). One EEG study found an increased activation of the parietal lobe in females, which indicated more spatially mediated strategies in females than males (19). Moreover, a study found that males tend to have more neuroanatomical activations than females in regards to neural activity during positive and negative emotion regulations. It was also found that the left dorsolateral, lateral orbitofrontal gyrus and right anterior cingulate gyrus were more activated in males than in females, who had only a strong activation of the left medial orbitofrontal gyrus (20). Males also indicated impaired inhibitory control after viewing erotic and painful stimuli, the effect that was not observed in females (21).

This study showed that the P300 and N200 latencies in the mid-central (Cz) and mid-parietal (Pz) were affected by the intensity of the arousal. Similar to previous findings, arousal intensity had an effect on the underlying neural substrates of emotion. That is, as expected, emotional arousal intensity was found to affect P300 and N200 latencies (in both the mid-central and mid-parietal regions). This effect was found mainly in regards to high arousal stimuli (images with an IAPS mean strength of 7–9) and low arousal stimuli (images with an IAPS mean strength of 1–3). This pattern was found in other studies as well (22). However, neutral stimuli indicated less effect for the arousal intensity. It was found that, only the mid-central (Cz) region of the brain (as indexed by N200) exhibited significant differences between neutral stimuli and high arousal stimuli albeit a weak one (*P* < 0.05). This inconsistency was found in other studies as well (13, 23–26). Moreover, significantly longer latency of high arousal intensity than low arousal intensity, perhaps, could be explained from the duration of reaction to positive and negative stimuli in response to high- and low-level of arousal images (27). In addition, it should be noted that such findings could have been affected by the fundamental mechanism of emotional arousal, which has a lengthening effect on the perception of emotion-processing durations (28).

## Conclusion

Sex is a fundamental factor in the interpretation of the elements of consciousness, attention and information processing in the course of emotion. In regards to the dimension of arousal, gender is a biological factor that shapes one’s psychological state especially the feeling of being awake or reaction to stimuli that range from calming to exciting. This finding has important implications for therapeutic intervention such as coma arousal procedure for head injuries and for increasing the effectiveness of related rehabilitation programs.

## Figures and Tables

**Table 1:** Amplitude and latency of P300 component

Variable/Outcome	Mean (Standard Deviation)							Interaction Effect	
	
	Male				Female			*F*	*P*-value
	
		High	Moderate	Low	High	Moderate	Low		
Amplitude	01	6.0 (4.06)	5.20 (3.82)	5.25 (3.29)	5.78 (3.46)	6.12 (3.64)	5.61 (3.41)	1.91	ns
	02	6.18 (4.01)	5.03 (3.00)	5.76 (3.13)	5.57 (3.55)	6.03 (3.60)	5.39 (3.20)	3.40	0.042
	Fz	3.25 (3.24)	3.26 (3.19)	2.70 (2.45)	2.76 (2.22)	2.49 (1.82)	2.46 (1.99)	0.45	ns
	Cz	3.14 (1.83)	2.05 (1.65)	1.71 (1.16)	2.74 (1.67)	2.04 (1.43)	1.70 (1.58)	0.57	ns
	Pz	4.65 (2.86)	3.89 (2.47)	3.83 (2.05)	4.76 (2.37)	4.69 (3.15)	3.62 (2.69)	1.50	ns
Latency	01	434.0 (102.9)	424.89 (116.27)	426.00 (120.48)	393.85 (107.06)	377.11 (100.27)	356.67 (65.98)	0.72	ns
	02	437.67 (123.07)	384.11 (105.56)	394.89 (93.53)	400.30 (111.00)	364.60 (84.93)	355.92 (63.23)	0.37	ns
	Fz	561.78 (119.33)	541.78 (132.41)	552.00 (144.39)	600.44 (108.14)	567.03 (129.10)	556.44 (140.29)	0.43	ns
	Cz	578.11 (98.52)	556.67 (98.79)	537.78 (121.05)	589.48 (99.71)	572.19 (103.88)	548.30 (104.74)	0.02	ns
	Pz	478.67 (122.86)	460.11 (116.69)	417.89 (113.94)	473.85 (111.93)	446.59 (116.07)	432.52 (105.15)	0.49	ns

ns: non-significant

**Table 2:** Amplitude and latency of N200 component

Variable/Outcome	Mean (Standard Deviation)							Interaction Effect	
	
	Male				Female			*F*	*P*-value
	
		High	Moderate	Low	High	Moderate	Low		
Amplitude	01	6.01 (5.31)	6.50 (4.74)	6.09 (4.42)	6.51 (3.97)	6.13 (3.87)	6.28 (3.79)	1.05	ns
	02	6.78 (4.20)	5.80 (3.96)	6.62 (4.52)	6.15 (3.59)	6.77 (4.25)	6.20 (3.59)	3.95	0.023
	Fz	2.36 (3.06)	2.72 (2.89)	1.90 (1.49)	2.61 (2.20)	2.25 (1.99)	2.41 (1.59)	2.16	ns
	Cz	1.63 (1.13)	1.80 (1.07)	1.22 (1.04)	2.08 (1.50)	1.62 (1.38)	1.92 (1.46)	3.10	ns
	Pz	4.56 (2.91)	3.56 (2.60)	4.04 (2.38)	4.33 (2.54)	4.48 (3.42)	3.66 (2.26)	3.00	ns
Latency	01	288.89 (70.35)	296.00 (63.03)	271.22 (59.39)	305.33 (65.29)	283.11 (69.06)	278.81 (61.57)	1.80	ns
	02	286.67 (72.75)	292.56 (69.21)	282.56 (58.23)	301.19 (63.67)	283.33 (66.39)	288.14 (58.64)	1.44	ns
	Fz	259.44 (64.22)	273.22 (76.12)	268.89 (65.40)	288.96 (55.85)	275.56 (71.51)	269.78 (65.63)	2.12	ns
	Cz	312.78 (51.72)	296.44 (48.47)	297.67 (48.20)	314.52 (50.96)	292.22 (53.06)	275.04 (48.79)	2.14	ns
	Pz	299.22 (67.15)	299.44 (60.38)	283.67 (54.91)	303.78 (55.31)	285.78 (58.19)	279.78 (64.12)	0.83	ns

ns: non-significant
